# Supplementary material for: Learning curve and surgical outcome of robotic assisted colorectal surgery with ERAS program
Source: Sci Rep. 2022 Nov 29;12:20566. doi: 10.1038/s41598-022-24665-w (PMC9709162; doi:10.1038/s41598-022-24665-w)
Supplement: Supplementary file 1 — Supplementary Information 1. [file 41598_2022_24665_MOESM1_ESM.zip › LSC Raw data-Ñ[▒K/073/N_ñw╝╨░O▒Kñσ.pdf]

W52 / W52

# 臺中榮民總醫院

[redacted]

## 基本資料

- 室別： 二醫手術室-43室，手術科別： CRS，手術類別： 常規，日期： 2020/03/19
- 進入時間： 1220 ； 麻醉開始時間： 1220 ； Time Out時間： 1300 ； 劃刀時間： 1301 ； 手術結束時間： 1650 送出時間： 1700 ；
- 麻醉方式： GE
  診斷：Malignant tumor of sigmoid colon
  手術名稱：Laparoscopic Anterior resection and anastomosis - malignant
  手術局部用藥：NIL
  術前預防性抗生素：
  - 抗生素名稱一： Veterin 1GM IVD/由麻醉人員給予
  - 時間： 1255
  - 上次簽章: 詹佳融

  ，追加預防性抗生素：
- 手術醫師： 蔣鋒帆，楊曙榮，陳映菁，江世偉
- 麻醉醫師： VS 賴慧卿
- 
  - 刷手護士A ： 阮鈺珊，時間： 1220-1500，接替刷手一： 朱詠暄，時間： 1500-1700
  - 流動護士A ： 王毓均，時間： 1220-1230，流動護士B ： 朱詠暄，時間： 1220-1500，接替流動一： 詹佳融，時間： 1230-1500，接替流動二： 阮鈺珊，時間： 1500-1700

---

## 皮膚狀況

A-V Shunt： 無
術前： 完整
　記錄者： 上次簽章: 詹佳融
術後： 完整
　記錄者： 上次簽章: 阮鈺珊

---

## 手術準備

手術臥式 單一手術臥式

- 
  - 手術臥式： 截石臥式
  - 使用附件： 溫毯 38 ℃，頭圈，腳架
  - 皮膚準備： 10%Aq Betadine，其它： 2% Cholhexidine
  - 電燒： 使用電燒: 電極片位置： 右大腿，記錄者： 上次簽章: 詹佳融

止血帶T： 無

---

## 植入物

- 植入物名稱/號碼/數量/部位：surgiwrap 0.02*100*130mm/壹/pelvic

，記錄者： 上次簽章: 阮鈺珊

---

## 標本送檢

- 細菌培養 無
- 細胞學 無
- 冰凍切片 無
- 病理標本
  組織名稱一：IMA LN
  ,
  組織名稱二：IMA ROOT
  ,
  組織名稱三：DISTAL CUT END
  ，更多組織名稱
  - 組織名稱四：SIGMOID RECTUM

- 截肢 無

標本交付 標本： 4 袋 , 標本： 0 盒 , 標本： 0 瓶 , 細胞學： 0 管 , Culture Tube： 0 管 , 被交付醫師： VS 蔣鋒帆 ,

- 記錄者： 上次簽章: 阮鈺珊
- 時間： 1630

## 傷口術後敷料

傷口敷料： 傷口敷料： 無

- 請於圖上標示部位：

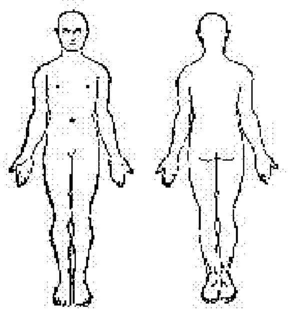

腹部切口

- 傷口敷料：mepore

## 導管

| 導管 | 天數 | 導管資訊 | 插入日期 | 插入時間 | 更換日期 | 拔除日期 | 拔除時間 | 拔除原因 | 預計拔除/更換 | 預計拔除/更換日期 |
|---|---|---|---|---|---|---|---|---|---|---|
| Foley(1) | 1 | 號碼:18 Fr.; 部位:膀胱; | 2020/03/19 | 1330 | | | | | | |
| Sump NG Tube(1) | 1 | 號碼:16 Fr.; 部位:胃; | 2020/03/19 | 1320 | | | | | | |
| Penrose Drain(1) | 1 | 號碼:12 mm.; 部位:骨盆腔; | 2020/03/19 | 1630 | | | | | | |
| Double-J(1) | 1 | 號碼:6X26 Fr.; 部位:輸尿管; 註記:右側; | 2020/03/19 | 1310 | | | | | | |
| Double-J(1) | 1 | 號碼:6X26 Fr.; 部位:輸尿管; 註記:左側; | 2020/03/19 | 1310 | | | | | | |

## 用物計數

- 手術中出血量： MINIMAL c.c.
- 手術中尿量： 600 c.c.
- 
  - 其它： 無

用物計數： 多切口同時縫合

- 1.器械： 正確 , 2.縫針： 正確 , 3.刀片： 正確 , 4.紗布： 正確 , 5.腹部墊： 未使用 , 6.紗球： 未使用 , 7.PEANUTS ： 未使用 , 8.紗條： 未使用 , 9.絲帶： 未使用 , 10.針頭： 未使用 , 11.Vessel loop： 未使用 , 12.Boots： 未使用 , 13.Jelco： 未使用 , 14.棉條： 未使用 ,
  - 第一次：
    - 刷手護士 ： 朱詠暄
    - 流動護士： 上次簽章: 阮鈺珊
    - 時間： 1600

,
  - 第二次：
    - 刷手護士 ： 朱詠暄
    - 流動護士： 上次簽章: 阮鈺珊
    - 時間： 1625

,
  - 第三次：
    - 刷手護士 ： 朱詠暄

- 流動護士： 上次簽章: 阮鈺珊
- 時間： 1640

## 備註

備註： NIL

- 記錄者： 上次簽章: 詹佳融
- 時間： 1330

填寫護理人員 阮鈺珊

正式摘要

W52 / W52

doctorRank:B
